# Quantification of energy input required for chitin nanocrystal aggregate size reduction through ultrasound

**DOI:** 10.1038/s41598-021-96657-1

**Published:** 2021-08-26

**Authors:** Ivanna Colijn, Remco Fokkink, Karin Schroën

**Affiliations:** 1 Wageningen University and Research, Food Process Engineering Group, Bornse Weilanden 9, 6708 WG Wageningen, The Netherlands; 2 Wageningen University and Research, Physical Chemistry and Soft Matter Group, Stippeneng 4, 6708 WE Wagningen, The Netherlands

**Keywords:** Engineering, Materials science, Nanoscience and technology

## Abstract

Nanoparticles have been claimed to contribute efficiently to e.g. the mechanical strength of composite materials when present as individual particles. However, these particles tend to aggregate. In this paper we prepare nanocrystals from chitin, a product with high potential added value for application in bio-based materials, and investigate the effect of ultrasound on de-aggregation. Chitin nanocrystals with a length ~ 200 nm and a diameter ~ 15 nm, were obtained via acid hydrolysis of crude chitin powder. Freeze drying resulted in severe aggregation and after redispersion sizes up to ~ 200 µm were found. Ultrasound treatment was applied and break up behaviour was investigated using static light scattering, dynamic light scattering, and laser diffraction. Our results suggest that the cumulative energy input was the dominant factor for chitin nanocrystal aggregate breakup. When a critical energy barrier of ~ 100 kJ/g chitin nanocrystals was exceeded, the chitin nanocrystal aggregates broke down to nanometre range. The break up was mostly a result of fragmentation: the aggregation energy of chitin nanocrystal aggregates was quantified to be ~ 370 kJ/g chitin nanocrystals and we hypothesize that mainly van der Waals interactions and hydrogen bonds are responsible for aggregation.

## Introduction

The versatile physical and chemical properties of nanoparticles give them outstanding properties for different applications^1^, including enhanced catalysis^2^, or drug release compared to their more macroscopic counterparts^3^. Also when embedded within a material, nanoparticles can alter the material’s properties such as the mechanical strength or give it antioxidant activity if the nanoparticles possess that property. A great example are carbon nanotubes for the production of flexible electronic devices or the use of bio-based polysaccharide nanocrystals which improve mechanical and barrier properties of polymeric materials^4–6^. These options to introduce unique properties make nanoparticles increasingly important as building blocks for different applications e.g. in the material, medical, and electronic science fields and industry.

Nanoparticles can be produced from various natural sources. Chitin, the second most abundant polysaccharide next to cellulose, is getting more and more attention^4,6,7^. It is present in cell walls of fungi, in insects, in marine sponges^8^, but mainly in exoskeletons of arthropods such as shrimps. The latter sources are currently considered waste materials produced by the fishery industry, but it could become the source for a high added-value product, because chitin can easily be extracted. Chitin is a polysaccharide composed of N-acetyl-2-amido-2-deoxy-d-glucoside units linked by β(1 → 4) bonds. The use of chitin can be expanded if the powder is hydrolysed into smaller chitin nanocrystals that have an increased exposed surface area in either solution or within a bulk material.

Chitin based nanofillers in particular posses special properties including a high aspect ratio, low density, and it was even reported that they retain their antimicrobial activity in polymeric matrixes^4,6,9–12^. In addition, their hydroxyl and amine groups allow surface modification, which can be used to tune nanoparticle properties practically at will, which is an important lead for further functionalisation. From this it is clear that chitin nanocrystals are versatile building blocks; in the current study we especially consider them as bio fillers in polymeric matrixes for the medical and food packaging industry.

For the envisioned application it is important to prevent degradation and reduce transportation costs, which can be achieved by drying. However, drying nanoparticles often leads to the formation of strong agglomerates because of its high surface area^13–18^, and in case of chitin nanocrystals due to the formation of strong hydrogen bonds^6^. Consequently, it remains difficult to redisperse the chitin nanocrystal aggregates in polymer melts, or in aqueous solutions depending on the application^7,19–22^. A common approach to facilitate nanoparticle dispersion is the use of surfactants and compatibilizers^13,15,16,23–25^. Alternatively, nanoparticles can be re-dispersed by the use of mechanical force e.g. ultrasound or extrusion. In contrast to extrusion, ultrasound has shown to effectively lead to a stable aqueous dispersion of nanoparticles^4^. Interestingly, this difference in dispersibility with treatment method has been observed for multiple nanoparticles such as cellulose nanocrystals^26,27^, or carbon nanotubes^28,29^.

Theoretically, aggregate break up occurs once the applied forces exceed the cohesive forces keeping the nanoparticles together. Aggregate break up can occur in two ways, i.e. fragmentation or erosion. Erosion is characterized by the removal of single or small parts from the parent aggregate, whereas fragmentation is characterized by the break up into pieces with similar sizes. Also the timescales of the two break up mechanisms are different as erosion occurs over much longer time scales compared to fragmentation^30^. To the best of our knowledge, the energy needed to break up chitin nanocrystal aggregates and their break up behaviour are unknown, yet for preparation of bio-based material reinforced with chitin nanocrystals these are essential design parameters.

The current study aims to quantify the aggregate energy of chitin nanocrystals and investigate its break up behaviour in terms of fragmentation and erosion. This will be investigated on a small scale by dispersing freeze dried chitin nanocrystal powder in Milli-Q water, and measuring particle size after ultrasound treatment by static light scattering, laser diffraction, and dynamic light scattering. We find a distinct transition in particle size as function of applied energy input. The data is compared to literature of polymer systems with chitin nanocrystals, and linked energy input in production systems.

## Materials and methods

### Materials

Shrimp chitin powder with > 98% purity and a high molecular weight was purchased from Glentham Life Sciences (United Kingdom). For dilutions only ultra-pure water was used (Milli-Q) (Millipore MilliQ system, Q-POD with Millipak Express 40 0.22 µm filter, Merck Millipore, USA).

### Sample preparation

Chitin nanocrystals were prepared via a slightly adjusted protecol of Broers et al.^31^. In short, chitin nanocrystals were prepared via acid hydrolysis of crude chitin powder in 3 M hydrochloric acid (HCl) at 85 °C for 90 min; 1 g of chitin powder per 15 ml HCl was added. The mixture was cooled on ice to stop the reaction, after which it was centrifuged at 2000*g* for 5 min (Sorvall LYNX 4000 superspeed centrifuge, Thermo Scientific™ 46910, MA, USA) to remove the HCl. The supernatant was discarded and an equal amount of Milli-Q water was added to redisperse the pellet. The latter step was repeated three times. Two final centrifugation steps were performed at 1000*g* for 5 min, after which the supernatant containing chitin nanocrystals was collected. After production, the 2.85 wt% chitin nanocrystal solution (pH ~ 2.0) was freeze dried at − 20 °C for at least 48 h (Christ Epsilon 2-6D Freeze Dryer, Martin Christ Gefriertrocknungsanlagen GmbH, Germany).

Dispersions of 0.01 wt% chitin nanocrystals in Milli-Q water (pH ~ 4.5) were prepared for static light scattering and dynamic light scattering experiments. Dispersions of 0.1 wt% chitin nanocrystals in Milli-Q water were prepared for laser diffraction and observations with fluorescent microscopy.

### Aggregate breakup by sonication

A Branson sonifier 250 connected to a 1/4 microtip (Branson Ultrasonics, United States) was used to sonify 10 ml sample at power 3, 5, 7, and 10 at a constant amplitude of 40%. This device had a horn frequency of 19,850–20,000 kHz. Samples were continuously cooled on ice to prevent excessive heating. The energy input (*E*_*input*_) was determined calorimetrically^32–33^ (Supplementary information 1):

1 $${E}_{input}= {C}_{p,water}* {m}_{water}*\frac{\Delta T}{\Delta t}$$

where *C*_*p*_ is the thermal capacity of water (4.18 J/K), *m* is the mass of water (0.2 kg) and Δ*T*/Δ*t* is the rise in temperature per time. Different power settings were used to differ the instantaneous power supplied; the measured instantaneous *E*_*inputs*_ were 5, 12, 18, and 32 J/s for the power settings 3, 5, 7, 10, respectively (Supplementary information 1). We enabled an *E*_*input*_ between 0 and 9590 kJ/g chitin nanocrystals. The heat loss to the environment was neglected because of the small volumes used.

### Characterization

#### Morphology

After acid hydrolysis, JOEL-JEM1400Plus-120 kV (spotsize 1) was used to observe the chitin nanocrystals, which were negatively stained in 2% uranylactate solution.

After ultrasound treatment, chitin nanocrystal dispersions were labelled with 0.01 wt% fluorescein isothiocyanate (FITC) for 24 h. The samples were centrifuged at 20,000*g*, after which the supernatant was discarded and an equal amount of Milli-Q water was added. The latter step was repeated five times. FITC grafting was confirmed with Fourier Transform Infrared (Bruker, Alpha II, Germany); FT-IR spectra were taken in absorbance mode over a wavenumber range of 400–4000/cm with a resolution of 4/cm and after 60 scan accumulations. The absence of FITC’s isothiocyanate characteristic peak (N=C=S strechting) at 2000/cm^34,35^ suggested that this group was involved in the reaction with the chitin nanocrystals (Supplementary Information 2). Afterwards, the samples were observed with Axioscope in fluorescent mode (Zeiss, Germany).

#### Degree of acetylation

^13^C cross polarization magnetic angle spinning (CP-MAS) NMR spectroscopy (Bruker Avance III HD spectrometer 700 MHz, Bruker, United States) was used to determine the degree of acetylation of the crude chitin powder and the produced chitin nanocrystals. Samples were packed into 4 mm zirconia rotors. The rotors were spun at MAS frequency of 11 kHz at 25 °C. The ^13^C CP MAS spectra were recorded with a recycle delay of 5 s, and contact time of 3 ms. The ^13^C NMR spectra were referenced with respect to adamantane (^13^C, 29.456 ppm). The degree of acetylation (DA%) was determined with the following equation^36^:

2 $$DA\%= \frac{{I}_{{CH}_{3}}}{{I}_{C1-C6}* \frac{1}{6}}$$

where *I*_*CH3*_ and *I*_*C1–C6*_ correspond the peak integrals associated to the CH_3_ and carbon backbone respectively. MestRenova software was used to determine the peak integrals.

As determined from the ^13^C NMR spectra (Supplementary Information 3), chitin nanoparticle production resulted in a slight decrease in degree of acetylation; a DA of 99% and 94% were found for crude chitin powder and the produced chitin nanocrystals respectively.

#### Thermal stability

The thermal stability of chitin powder and the produced chitin nanocrystals were determined with thermogravimetric analysis (TGA) (PerkinElmer TGA 4000, Waltham, MA, USA). The samples were heated from 30 to 450 °C, at a heating rate of 10 °C/min under a constant nitrogen flow of 20 mL/min. Pyris software was used to examine the data (Pyris, 11.1.1.0492). For chitin powder an initial degradation temperature and maximum temperature of 216 °C and 334 °C were found respectively. For chitin nanocrystals an initial degradation and maximum degradation temperature of 148 °C and 260 °C respectively.

#### Particle size distribution

The particle size distributions of the chitin nanocrystal dispersions were measured with laser diffraction (Mastersizer 3000, Malvern Instruments Ltd., United Kingdom) and dynamic light scattering (Zetasizer Ultra, Malvern Instruments Ltd, United Kingdom). The absorption index was set to 0.01, and a refractive index of 1.560 and 1.330 was used for chitin nanocrystals and Milli-Q water, respectively. All samples were measured in triplicate.

**Figure 1:**
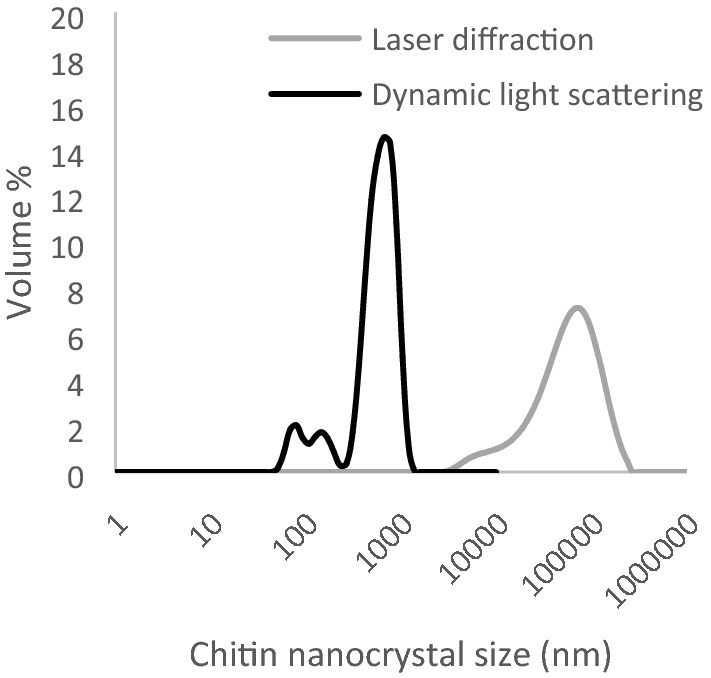
Volume % as function of chitin nanocrystal size at an *E*_*input*_ of 0 kJ/g chitin nanocrystals measured with laser diffraction and dynamic light scattering. The average of three measurements is given.

Please note that the measurement angle used for laser diffraction (0.015°–144°) and dynamic light scattering (173°) was different. Consequently, as mainly forward scattering was used for laser diffraction, it was more sensitive toward particles with a size above the wavelength of the device laser (i.e. *λ*_*device*_ = 632.8 nm for the red source and λ_device_ = 470 nm for the blue source). As back scattering was used during dynamic light scattering measurements, also particles with a size below λ_device_ could be observed (i.e. *λ*_*device*_ = 632.7 nm). Because of the very polydisperse nature of our chitin nanocrystal aggregate sample, we find combination of these techniques crucial to obtain a good overall impression of the size distribution (Fig. 1).

#### Static light scattering

A HeNe 2 mW 633 nm polarized laser (product 19064, LASOS, United States) was shone through 8 mL 0.01 wt.% chitin nanocrystal dispersion which was added to the small angle light scattering cell (Anton Paar, Austria). The incoming beam was blocked by an inhouse made beam stop, that was placed on a ground glass diffuser (gritt 600, Edmund Optics, United States). The distance between the SALS cell and the ground glass diffuser was 31 cm. A charged coupled device camera (CCD Thorland 125 IM SERIES, Edmund Optics, United states) with a lens of 16 mm/F1.4 59879 (Edmund Optics, United States) was used to capture 50 images per sample. The scattering patterns were further analyzed with Fiji^37^ (Supplementary Information 4). The Radial Profile Extended plugin developed by Baggethun^38^ was used to derive the scattering intensity as function of scattering path (Supplementary Information 5). The total scattering intensity is defined as the integral of the scattering intensity as function of the scattering path, which was corrected for the background intensity (Supplementary Information 5). The first 120 pixels of the path lengths were not considered as this corresponded to the position of the beam stop.

## Results

Application of ultrasound has shown to be an effective way to break down nanoparticle aggregates^4,26,28^, and was for that reason used to quantify the aggregation energy (*E*_*aggregate*_) within chitin nanocrystal aggregates. The energy input (*E*_*input*_) produced by ultrasound was determined calorimetrically for different sonication power settings, enabling an *E*_*input*_ up to 9650 kJ/g chitin nanocrystals (Supplementary Information 1).

### Morphology of chitin nanocrystals and their aggregates after ultrasound

**Figure 2:**
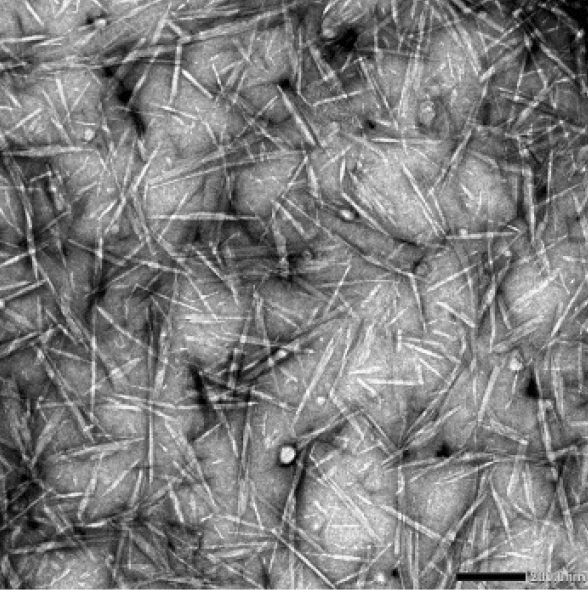
Needle shaped chitin nanocrystals derived after acid hydrolysis observed with transmission electron microscopy. The scale bar has a size of 200 nm.

The morphology and size of the individual chitin nanocrystals and their aggregates was observed with transmission electron microscopy and fluorescence microscopy, respectively. Figure 2 shows the morphology of individual chitin nanocrystals that have a clear needle like morphology. The chitin nanocrystals had a length between 50 and 400 nm and a diameter between 10 and 20 nm. The geometry of the nanocrystals corresponded well with sizes found in literature^4,6,7,31^. In literature, chitin nanocrystals commonly show a crystallinity index of 85–90%^4,39,40^.

**Figure 3:**
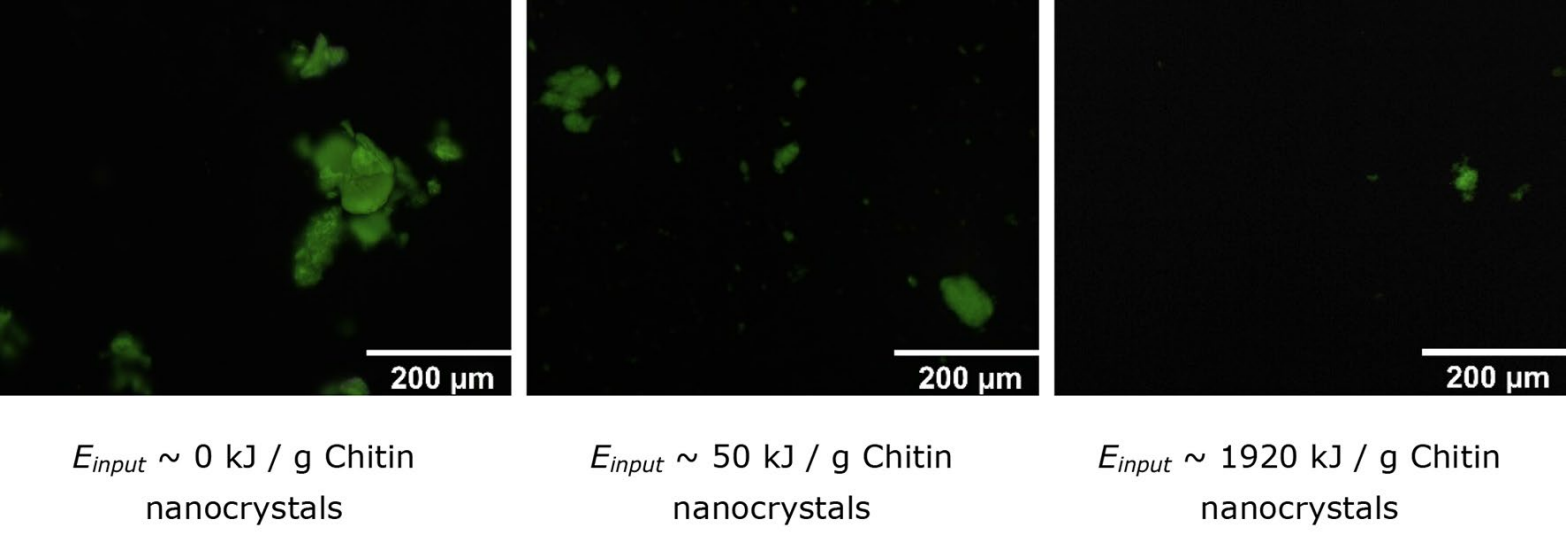
Microscopic pictures of FITC labelled chitin nanocrystal particles after sonication treatments at different *E*_*input*_.

After ultrasound treatment, the chitin nanocrystal aggregates were labelled with FTIC to enable observation with fluorescent microscopy (Fig. 3). After freeze drying, aggregates with sizes up to 200 µm were observed in the chitin nanocrystal dispersion that was very polydisperse. Ultrasound clearly decreased the chitin nanocrystal aggregates; at an *E*_*input*_ ~ 1920 J/g chitin nanocrystal hardly any aggregates were visible, and if visible they had a size < 40 µm. As the resolution of the microscopy is ~ 2 µm, this probably suggests that most chitin nanocrystal particles were smaller than that, assuming no reduced signal intensity as function of size.

### Aggregate strength

**Figure 4:**
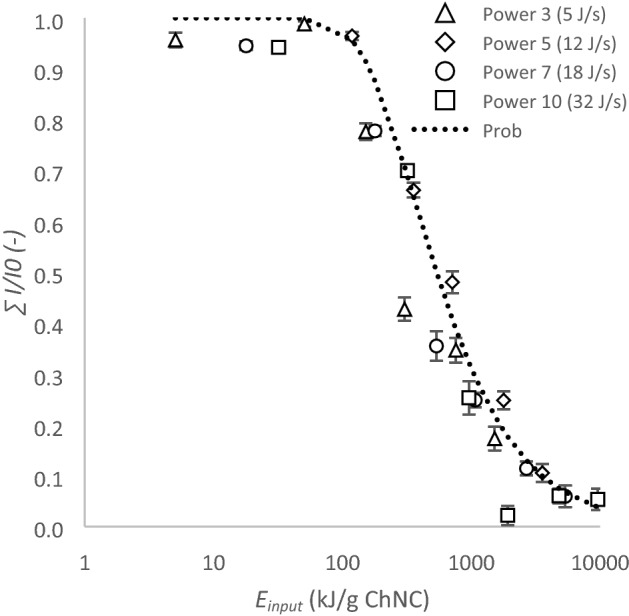
Normalized total intensity as function of ultrasound energy (*E*_*input*_) in kJ/g chitin nanocrystals determined via static light scattering.

Static light scattering was used to capture the overall aggregate break up behavior. According to the classical Raleigh scattering theory a *I* ~ *r*^6^ relationship exists, meaning that the total scattering intensity decreases when an aggregate breaks up into two smaller particles of the same total volume. The scattering intensity clearly decreased as a consequence of ultrasound treatment (Supplementary Informations 4 and 5) and this is summarized in Fig. 4, showing the normalized total scattering intensity as function of *E*_*input*_ produced by ultrasound.

The effect of ultrasound on scattering intensity could be divided into three regimes. In the first regime < 100 kJ/g chitin nanocrystal, no effect of *E*_*input*_ on scattering intensity was observed. In the second regime, 100 kJ/g chitin nanocrystal < *E*_*input*_ < 5000 kJ/g chitin nanocrystal, the total intensity decreased as a consequence of either a decreased particle size, or decreased number of aggregates, but most likely a combination of both. At *E*_*input*_ > 5000 kJ/g chitin nanocrystal, the scattering intensity was very close to the background intensity and did not decrease any further.

The dimensionless number Prob has been suggested to determine the breakup probability of an aggregate at a certain shear rate^41–43^:

3 $$Prob={e}^{-\sigma /\tau }$$

where *σ* is the mechanical bonding strength of an aggregate in N/m^2^ and *τ* represents the shear stress in N/m^2^. A slightly modified version was used in the current study. To describe the breakup probability of an aggregate at a certain *E*_*input*_, *σ* and *τ* were replaced with *E*_*aggregate*_ and *E*_*input*_, respectively. In addition, the mirrored value was taken to fit the equation to the static light scattering data points:

4 $$Prob=1- {e}^{\left(\frac{{E}_{aggregate}}{{E}_{input}}\right)}$$

From the fit of Eq. (4) to the static light scattering data points, an *E*_*bond*_ of 373 kJ/g chitin nanocrystal was derived, that is put into a wider perspective in the discussion section.

### Particle size distribution

**Figure 5:**
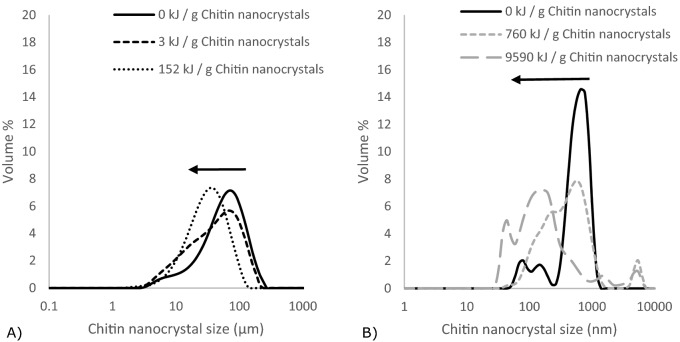
The volume % as function of chitin nanocrystal size at different *E*_*input*_ measured with (**A**) laser diffraction and (**B**) dynamic light scattering.

**Figure 6:**
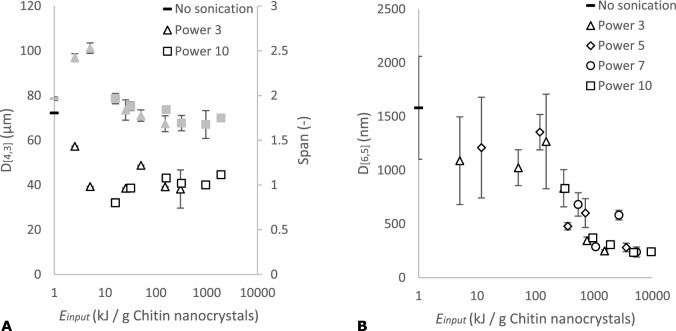
The chitin nanocrystal size (in white) and span (in grey) as function of ultrasound *E*_*input*_ at different power settings measured with (**A**) laser diffraction and (**B**) dynamic light scattering. The error bars represent the standard deviation within three different measurements, some of them being within the data marks.

To distinguish between aggregate break up occurring in particles of different size, size distributions after ultrasound treatment were measured using different techniques. Figure 5 shows size distributions obtained with laser diffraction and dynamic light scattering. The size averages are given as D_[4, 3]_ for laser diffraction and D_[6, 5]_ for dynamic light scattering as function of *E*_*input*_, and are not the same (Fig. 6).

Chitin particle sizes between 5 and 500 µm and 50–100 nm were obeserved with laser diffraction and dynamic light scattering, respectively. We interpret this as follows: only a very small number of large particles (> 5 µm) was present, and these particles scatter mostly in the forward direction and are dominant at low angles. At a larger detection angle as used for dynamic light scattering, their contribution is negligible and does not contribute to the overall signal. If there would have been many large particles, they would have given a signal during this measurement, and that is not the case, not even at tenfold higher concentration. Thus, the overall behavior is dominated by break-up events happening in small(er) chitin aggregates, and this was well captured by static light scattering (Fig. 4).

On a more general level, at an *E*_*input*_ < 16 kJ/g the chitin nanocrystal size was reduced from 72 µm to approximately 40 µm (Fig. 6), leading to higher polydispersity. This effect was supported by results obtained by dynamic light scattering; the chitin nanocrystal size shifted to lower values and broader distributions (*E*_*input*_ ~ 3 kJ/g chitin nanocrystals) (Fig. 5). At 16 < *E*_*input*_ < 100 kJ/g chitin nanocrystals, no further decrease in chitin nanocrystal size was observed, and the span remained equal, which corresponds well with the static light scattering results (Fig. 4). At higher *E*_*input*_ values, most of the chitin nanocrystal aggregates broke up to a size of ~ 240 nm which is similar to the length of the original chitin nanocrystals before freeze drying (Fig. 2). Although ultrasound treatment clearly shifted the chitin nanocrystal size to lower values, also when no ultrasound treatment was applied, chitin nanocrystal particles in the nano range were found, and at the highest *E*_*input*_, the chitin nanocrystal particles showed a considerable size distribution (Fig. 6).

## Discussion

Ultrasound treatment clearly reduced the chitin nanocrystal aggregate size (Figs. 3 and 6), as was found for different nanoparticles including carbon nanotubes^28^ and cellulose nanocrystals^26^. Our results suggest that weakly bound micro meter agglomerates can be broken down at low *E*_*input*_ < 16 kJ/g chitin nanocrystals (Fig. 6), which corresponds to the general observation that break up occurs at the weakest spot inside the aggregate. However, a much higher critical *E*_*input*_ of ~ 100 kJ/g chitin nanocrystals was required to decrease the aggregates to the size range of the original chitin nanocrystals (Figs. 4 and 6). This is common for particles, and far from trivial for nanoparticles because the *E*_*input*_ required increases as the particle diameter decreases^44^.

An aggregation energy (*E*_*aggregate*_) of ~ 370 kJ/g chitin nanocrystals was found (Fig. 4). We would like to emphasize that this number corresponds to the break up of interparticle interactions within an aggregate, rather than the interactions within a nanocrystal. It is good to point out that extreme sonication conditions, e.g. 300 W for 30 min, are capable of separating chitin nanofibrils from the chitin matrix but are not able to break the nanofibrils themselves^45–47^. It is expected that mainly van der Waals interactions and hydrogen bonds formed after drying are responsible for the high aggregation strength^47,48^. Interestingly, these latter interactions are also believed to be responsible for keeping individual chitin polymers within a chitin nanocrystal together^49^, although the actual strength can be different. Within a nanocrystal, chitin polymers have an extremely evolved hierarchical structure^49^, which results in a strong material built by relatively weak interactions, i.e. van der Waals interactions (~ 1* k*_*b*_*T*) and hydrogen bonds (~ 10 *k*_*b*_*T*)^50^. In a freeze dried sample irregularly aggregates are present (Fig. 3), that likely do not have as many interactions as the chitin nanocrystals would have, which explains the differences in between both materials.

We expect these high *E*_*input*_ values needed to break up aggregates to be one of the reasons why it remains difficult to achieve homogeneous chitin nanocrystal distributions in polymeric matrixes without any surface modification or the use of a compatibilizer. Extrusion is often used to process thermoplastic polymers, where typical specific mechanical energy inputs lay in the range of 0.17–0.27 kWh/kg. Considering a maximum residence time of 10 min and a chitin nanocrystal content of 5 wt%, an *E*_*input*_ of around ~ 1 kJ/g material would be achieved. Assuming *E*_*input*_ is equally distributed through the whole material, ~ 0.05 kJ/g chitin nanocrystals is available for aggregate break down, being lower than the critical energy barrier of ~ 100 kJ/g chitin nanocrystals that we identified before. Thus, in this example the *E*_*input*_ provided by extrusion should be increased by at least a factor 2000. Even if we consider that not all acoustical energy is transferrred into cavitation breaking energy (~ 35% according to ^33^), serious increase in *E*_*input*_ is required for break up to occur. This difference becomes more pronounced using the argument of maximum local shear stress that can be achieved. Huang and Terentjev calculated a local shear stress of 20 kPa for mechanical shear mixing in high viscosity polymer melts, whereas 100 MPa could be achieved for ultrasound treatment in low viscosity solvents; this is a factor 5000 different^28^. Please note that in the latter study it was assumed that all stress from an imploding bubble contributed to the localized shear stress.

The dominant break up mechanism is expected to be fragmentation as deduced from the various size distributions (Figs. 5 and 6), which also corresponds well with observations of others^26,51,52^. Like Graves et al.^52^ we find that the *E*_*input*_ was the dominant factor for nanoparticle aggregate break up. However, the reported dependency on *E*_*input*_ is not always observed^26^. This may be related to the calculation of the energy input through the implosion of a bubble in case of Beuguel et al.^26^, and determined calorimetrically by Graves et al.^52^ and the current study. The reasons for this strong dependency on *E*_*input*_ are not yet elucidated.

As the next step toward application in e.g. polymer melts other factors like interfacial compatibility should be considered as well. When relatively hydrophilic chitin nanocrystals are added to hydrophobic matrixes, there will be a continuous competition between the hydrodynamic forces breaking the chitin nanocrystal particles up and the cohesive forces bringing the chitin nanocrystal particles together. This is commonly observed for nanoparticles in polymer and aqueous systems^26,53–55^. So ways to reduce *E*_*aggregate*_, away from the use of compatibilizers, are relevant. Interestingly, *E*_*aggregate*_ does not solely depend on the interaction forces, for example, Khoshkava and Kamal^17^ found that cellulose nanocrystal aggregates with a more porous structure require less energy to break up. Van der Waals interactions act over a longer range (0.32 nm) compared to hydrogen bonds (up to 100 nm), thereby explaining why less *E*_*input*_ is needed to break down larger nanoparticles compared to smaller ones. Higher porosity can be achieved using other drying methods such as spray drying or by using lower chitin nanocrystal concentrations during freeze drying^17,56^. Another way to decrease the *E*_*input*_ required for chitin nanocrystal break up, would be to increase the interfacial compatibility with the solvent or polymer melt. This can be achieved amongst others by surface modification which is part of our follow-up research.

## Conclusion

We have demonstrated that the critical energy barrier for aggregate break-up as well as aggregation energies can be quantified with a combination of ultrasound and static light scattering. Ultrasound treatment was shown to effectively decrease the size of chitin nanocrystal aggregates that were held together by van der Waals interactions and hydrogen bonds (~ 370 kJ/g chitin nanocrystals) formed during freeze drying. The reason for the strong relationship between the cumulative applied energy input and the break up behavior of the chitin nanocrystals is not yet elucidated.

Although ultrasound can easily overcome the critical energy input needed to break up chitin nanocrystal aggregates, the energy input achieved during extrusion of polymer melts is expected to be too low to achieve this, which is in line with the common observation that nanoparticles do not disperse well in polymer melts. Still, the current study very clearly sheds light on the importance of energy input as a design parameter for nanocomposite preparation, and also directs toward which strategies should be applied to achieve nanoparticle dispersion (e.g. surface modification).

## Supplementary Information

Supplementary Information.
