# Supplementary material for: Quantification of energy input required for chitin nanocrystal aggregate size reduction through ultrasound
Source: Sci Rep. 2021 Aug 26;11:17217. doi: 10.1038/s41598-021-96657-1 (PMC8390482; doi:10.1038/s41598-021-96657-1)
Supplement: Supplementary file 1 — Supplementary Information. [file 41598_2021_96657_MOESM1_ESM.docx]

# **Supplementary Information**

**Supplementary Information 1:** The sonication energy in kJ s^-1^ for different ultrasound power settings determined calorimetrically.

| \| 2000 cm^-1^ \| \| --- \| \| **Supplementary Information 2:** FTIR spectra of FITC, chitin nanocrystals (ChNC) and FITC labelled chitin nanocrystals (ChNC-FITC). The absence of the characteristic peak of FITC’s isocyanate group at 2000 cm^-1^ confirms the conjugation of FITC with the amine groups of the chitin nanocrystals. \|   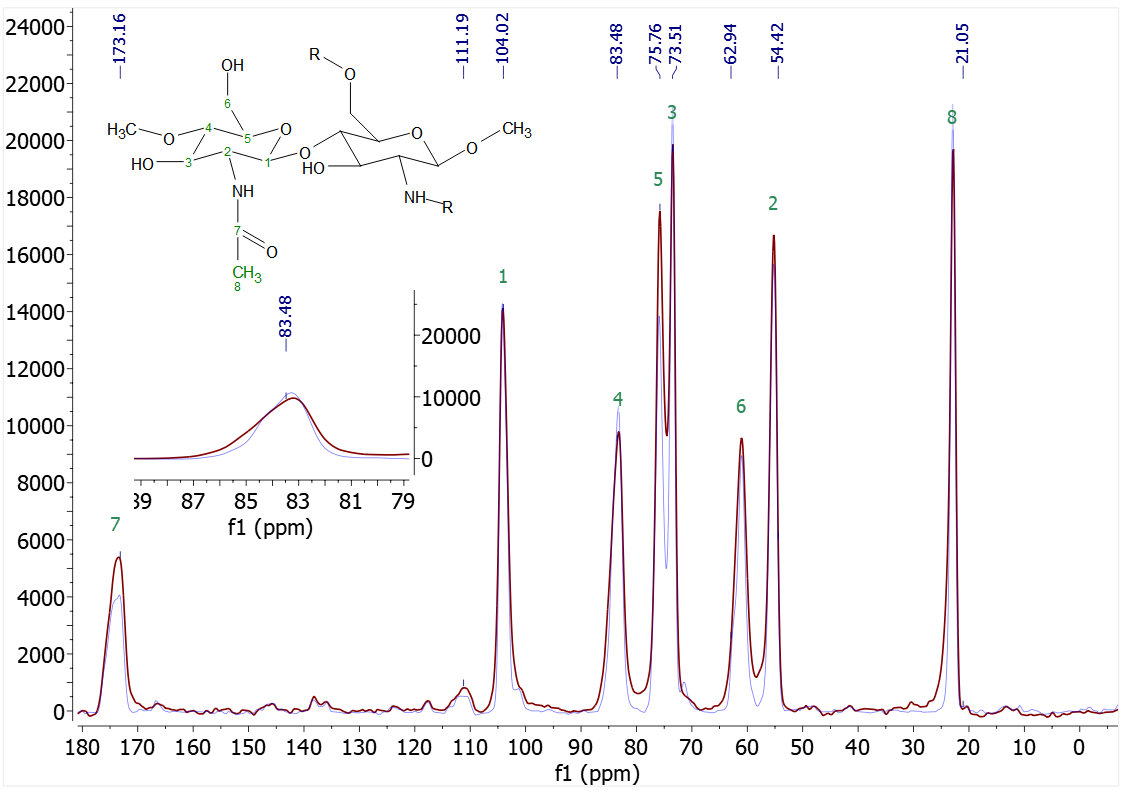 |
| --- | --- | --- |
| **Supplementary Information 3:** ^13^C NMR spectra of crude chitin powder (red) and produced chitin nanocrystals (blue). The green numbers indicate the peaks that were asisgned to their corresponding carbon atoms as represented in the chemical drawing. |

| 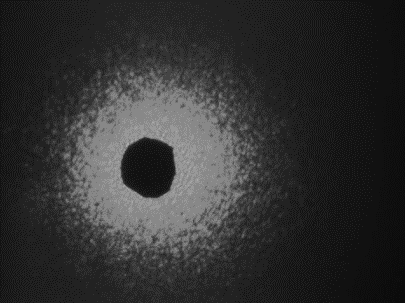 | 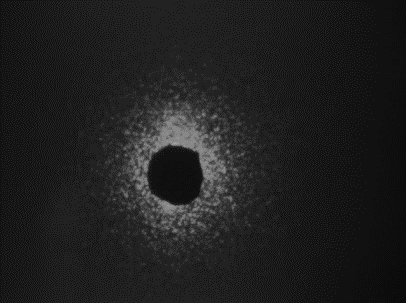 | 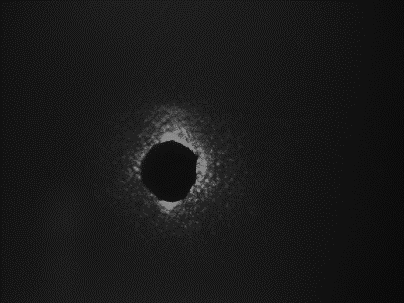 |
| --- | --- | --- |
| A) | B) | C) |
| **Supplementary Information 4:** Scattering patterns derived with static light scattering A) at an *E_input_ ~* 0 J / g Chitin nanocrystals B), an *E_input_* ~ 537 kJ / g Chitin nanocrystals C) an *E_input_* of ~ 9590 kJ / g Chitin nanocrystals | | |
|  | | |
| **Supplementary information 5:** Scattering intensity as function of scattering path at different *E_input_* before background correction and normalization. | | |
